# Queuosine-modification dependent histidyl tRNA fragment promotes cell-type dependent proliferation

**DOI:** 10.1093/nar/gkag1101

**Published:** 2026-10-31

**Authors:** Wen Zhang, Brandon J Chew, Zaneta Matuszek, Sihao Huang, Luke R Frietze, Hoang Anh V Tran, Christopher D Katanski, Marcus Foo, Qing Dai, Tao Pan

**Affiliations:** Department of Biochemistry and Molecular Biology, University of Chicago, Chicago, IL 60637, United States; Department of Molecular Genetics and Cell Biology, University of Chicago, Chicago, IL 60637, United States; Department of Biochemistry and Molecular Biology, University of Chicago, Chicago, IL 60637, United States; Department of Biochemistry and Molecular Biology, University of Chicago, Chicago, IL 60637, United States; Department of Biochemistry and Molecular Biology, University of Chicago, Chicago, IL 60637, United States; Department of Biochemistry and Molecular Biology, University of Chicago, Chicago, IL 60637, United States; Department of Biochemistry and Molecular Biology, University of Chicago, Chicago, IL 60637, United States; Committee on Microbiology, University of Chicago, Chicago, IL 60637, United States; Department of Biochemistry and Molecular Biology, University of Chicago, Chicago, IL 60637, United States; Department of Biochemistry and Molecular Biology, University of Chicago, Chicago, IL 60637, United States

## Abstract

tRNA fragments (tRFs) are natural, small non-coding RNAs produced by endogenous ribonucleolytic cleavage of tRNAs. tRFs play a role in many biological pathways, including regulation of cell proliferation. Specific tRNA modifications affect tRF biogenesis. Queuosine (Q) modification occurs at the wobble anticodon nucleotide of 4 mammalian tRNAs and protects these tRNAs from fragmentation. Here, we investigated cell-type and tRNA Q-modification dependencies on mammalian cell proliferation. We found that Q-modified tRNA can either increase, decrease, or make no difference in proliferation. Proliferation decreased in MCF7, a breast cancer cell line, when tRNAs were Q-modified and dependent on the modification enzyme complex, QTRT1/2. MCF7 proliferation increases upon the transfection of Q-modification-dependent tRF^His^ or decreases upon knockdown of endogenous tRF^His^. Consistent with Q-dependent proliferation reduction, polysome profiling showed decreased translation efficiency of ribosomal proteins. Finally, protein pull-down of MCF7 lysates using tRF^His^ identified Musashi RNA-binding protein 2 (Msi2) as a tRF^His^-binding protein. Msi2 knockdown reduced the Q-modification-dependent or tRF^His^ dependent cell proliferation effect. Our results illustrate a highly cell-type-dependent proliferation effect of tRNA Q-modification, suggesting multi-faceted mechanisms in studying this ubiquitous tRNA modification.

## Graphical Abstract

**Graphical Abstract:**
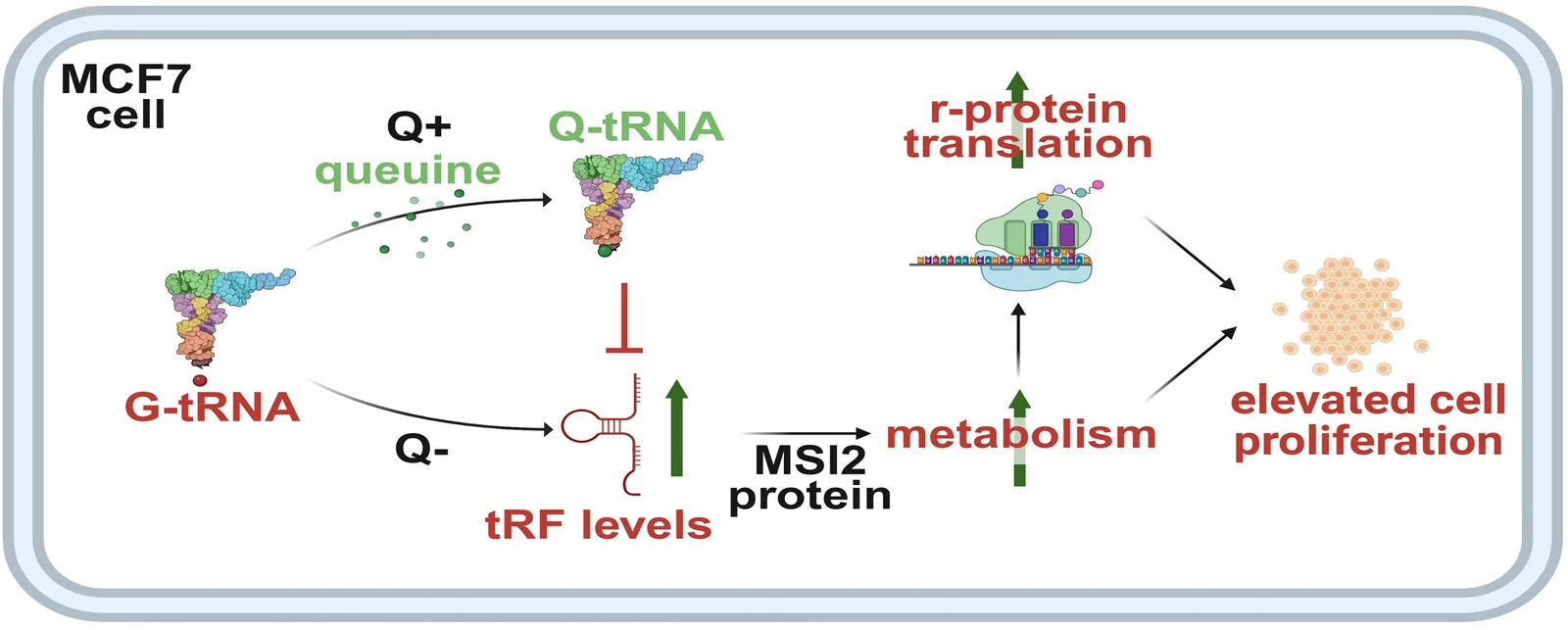

## Introduction

Among the over 150 distinct chemical modifications in biological RNAs [1], eukaryotic transfer RNAs (tRNAs) are the more decorated, at an average of 13 modifications per molecule [2]. tRNA modifications affect many aspects of tRNA function, such as decoding efficiency and accuracy, stability, aminoacylation, and more [3–8]. Among these, queuosine (Q, Supplementary Fig. S1A) is a unique eukaryotic tRNA modification, as its biogenesis is completely dependent on gut microbes and/or diet in metazoans [9]. Q-modification occurs at the wobble G34 anticodon position of tRNAs of Tyr/His/Asn/Asp. In the human gut bacteria, tRNA Q-modification is synthesized *de novo* through an 8-step biosynthesis pathway starting with GTP [9, 10]. Upon bacterial turnover, Q-modified tRNA is catabolized to the nucleobase queuine, which is imported by human cells [11], and incorporated onto cytosolic and mitochondrial tRNAs by the enzyme composed of the human proteins QTRT1 and QTRT2 [9, 12, 13].

Although shown to be non-essential in mice [14], Q can increase the efficiency of tRNA acylation [15], impart codon bias in bacteria, fruit flies, and mice [16–18], and alter translational fidelity [19]. Also, Q-modified tRNAs can accumulate in brain tissue [20] and impact synaptogenesis and neuronal morphology in a sex-dependent manner in mice [18], providing further evidence of crosstalk between gut microbes and brain.

Q-modification also protects their cognate tRNAs from endogenous fragmentation [21]. tRNA fragments (tRFs) are cellular endoribonucleolytic cleavage products of both nascent and mature tRNAs [22, 23]. tRFs can affect many aspects of a cell. For example, they can be loaded onto Argonaute (Ago) proteins to guide silencing of certain genes [24, 25], or sequester RNA-binding proteins to promote degradation of specific mRNA transcripts [26]. Furthermore, tRFs have been shown to be units of “epigenetic inheritance” as they are packaged into sperm to silence retroviral elements [27, 28], indicating that tRNA fragments can serve many purposes during specific phases of development. At the cellular level, sex-hormone dependent tRNA-derived RNAs (SHOT-RNAs) are specifically produced in androgen receptor (AR)-positive and estrogen receptor (ER)-positive cancer cells [29]. These SHOT-RNAs were found to have a significant impact on cell proliferation and tumorigenesis.

An understudied question is how tRNA Q-modification affects cell proliferation in a cell-type-dependent manner. There are a wide range of studies [30–33] reporting Q-modification increasing, decreasing, or having no effect on mammalian cell proliferation. These inconsistent results were obtained from studies by different labs in separate studies. Furthermore, a common trend in the literature is to assume that increases in Q-modification analogously increase proliferation, resulting in little mechanistic investigation into how Q-modification may actually slow down proliferation.

Here, we carry out a cell-type study of tRNA Q-modification effect on cell proliferation and provide a mechanistic underpinning for how Q-modification can slow proliferation. We have previously shown that tRNA Q-modification can increase proliferation of HEK293T and mouse bone marrow dentritic cells [34]. Among the six cell lines tested here—HeLa, MEF, HCT116, MCF10A, MDA-MB-231, and MCF7—only MCF7, an ER+ breast cancer cell line, exhibits slower proliferation when tRNAs are Q-modified. We found that MCF7 cells without Q-modification produce higher levels of fragments of Q-modifiable tRNA^His^ (tRF^His^). The slow proliferation effect is alleviated upon tRF^His^ transfection or exacerbated upon knockdown of endogenous tRF^His^. Mechanistic studies using tRNA-seq that simultaneously examine Q-modification-dependent tRNA abundance and modification, along with mRNA-seq of polysome profiles, show alterations in tRNA properties and codon usage patterns that are consistent with increased proliferation when tRNAs are not Q-modified. Additionally, we identify Musashi RNA binding protein 2 (Msi2) as a tRF^His^ binding protein. The tRF^His^-dependent proliferation is reduced upon Msi2 protein knockdown, suggesting a direct protein target for the cellular tRF^His^ activity in MCF7 cells.

## Materials and methods

### Cell culture and generation of Q± and Q− cells

To generate Q+ and Q− cells, cell culture was performed similarly to a previous study [21]. Cells were grown at 37°C in a CO_2_ incubator with Dulbecco’s modified Eagle’s medium (DMEM), 10% dialyzed fetal bovine serum (ThermoFisher, 26400044), and 1% penicillin–streptomycin to generate Q-depleted cells (Q−). For MCF7 cells, 10 µg/ml bovine insulin, and 10 nM beta-estradiol were added to the above cell media (Invitrogen, Carlsbad, CA, USA). To restore Q (Q+), 1 µM of queuine was added and incubated for at least 24 h, each time the cells were passaged.

Dialyzed FBS was obtained from Thermo Fisher Scientific/Gibco (Catalog No. 26400044). According to the manufacturer, the serum is dialyzed by tangential flow filtration against 0.15 M NaCl using a 10 000 molecular weight cutoff membrane until the glucose concentration is <5 mg/dl. This process depletes low-molecular-weight dialyzable components, including glucose, amino acids, small peptides, hormones, and other small exogenous metabolites. Because queuine is a small molecule (∼277 Da), it is expected to be substantially depleted from dialyzed FBS.

### Cellular fractionations

Cytoplasmic and nuclear fractions were separated using NE-PER (ThermoFisher) based on the manufacturer’s protocol.

### Pulldown mass spec

The following 3′ biotinylated-oligos were used for pulldowns:

His5p-biotin: 5′-pGGCCGUGAUCGUAUAGUGGUUAGU ACUCUGCGUU-biotin

His5pSC-biotin: 5′-pGGUAGCGAAGCCUUUCGACGUU UGGUCUUGAUGUU-biotin

∼270 pmols of each biotinylated oligo were denatured at 90°C for 1 min and incubated at room temperature for 5 min. 20 mM Tris–HCl pH 7.5, 2 mM MgCl_2_, and 400 mM KCl (renaturation buffer) was added to the oligos and incubated at 37°C for 5 min. Nuclear and cytoplasmic extracts were added to the oligos and rotated for 2 h at 4°C for binding. One milligram of streptavidin beads (ThermoFisher) were added to 1 mg of total protein extract. The solution was removed and the beads were washed with 900 µl of wash buffer (10 mM Tris–HCl pH 7.5, 100 mM NaCl, 150 mM KCl, 1.5 mM MgCl_2_, 0.05% v/v IGEPAL CA-630). Proteins were incubated with a high-salt elution buffer (1 M NaCl, 10 mM Tris–HCl pH 7.5, 100 mM NaCl, 150 mM KCl, 1.5 mM MgCl_2_, 0.05% v/v IGEPAL CA-630), vortexed for 30 s, and incubated for 5 min at 37°C. Elutions were loaded onto an SDS–PAGE gel, and relevant bands were cut out and sent to the University of Nebraska–Lincoln for analysis with nanoLC-MS/MS. According to Proteomics and Metabolomics Facility’s protocol, samples were trypsinized overnight at 37°C and Peptides were extracted from the gel pieces, dried down, and re-dissolved in 2.5% acetonitrile, 0.1% formic acid. Each digest was run by nanoLC-MS/MS using a 1-h gradient on a 0.075 mm × 250 mm C18 column feeding into a Q-Exactive HF mass spectrometer. All MS/MS samples were analyzed using Mascot (Matrix Science, London, UK; version 2.6.1). Mascot was set up to search the cRAP_20150130.fasta; uniprot-human_20180625 database (73 162 entries), assuming that the digestion enzyme was trypsin. Mascot was searched with a fragment ion mass tolerance of 0.060 Da and a parent ion tolerance of 10.0 PPM. Deamidation of asparagine and glutamine and oxidation of methionine and carbamidomethyl of cysteine were specified in Mascot as variable modifications. Scaffold (version Scaffold_4.8.4, Proteome Software Inc., Portland, OR) was used to validate MS/MS based peptide and protein identifications. Peptide identifications were accepted if they could be established at greater than 80.0% probability by the Peptide Prophet algorithm [35] with Scaffold delta-mass correction. Protein identifications were accepted if they could be established at greater than 99.0% probability and contained at least 2 identified peptides. Protein probabilities were assigned by the Protein Prophet algorithm [36]. Proteins that contained similar peptides and could not be differentiated based on MS/MS analysis alone were grouped to satisfy the principles of parsimony. Proteins sharing significant peptide evidence were grouped into clusters.

### Polysome profiling

Polysome profiling was performed as previously described [37]. Fifteen-centimeter plates of MCF7 cells at ∼60% confluency were treated with 100 µg/ml cycloheximide (CHX) for 7 min at 37°C. Cells were washed twice with ice-cold 1× PBS with 100 µg/ml CHX. Cells were collected in 5 ml of ice-cold 1× PBS with 100 µg/ml CHX and pelleted at 1000 × *g* for 5 min. Cell pellets were resuspended in 3–4 volumes of lysis buffer [20 mM HEPES, pH 7.6, 100 mM KCl, 5 mM MgCl_2_, 1% Triton X-100, 100 µg/ml CHX, freshly added 1× protease inhibitor (11873580001, Roche), 40 U/µl RNase inhibitor (AM2696, ThermoFisher)] and incubated on ice for 20 min with periodic perturbation. Samples were centrifuged at 16 000 × *g* for 15 min at 4°C and the clear supernatant was collected. Four microliters of Turbo DNase was added to the supernatant and incubated at room temperature for 15 min (AM2238, ThermoFisher). A 5%–50% sucrose gradient (20 mM HEPES, pH 7.6, 100 mM KCl, 5 mM MgCl_2_, 100 µg/ml CHX, fresh 1× protease inhibitor, 40 U/µl RNase inhibitor) was prepared using a Biocomp gradient station. About 600 µl gradient buffer was removed from the top of the sucrose gradient. An equal amount of cell lysate, usually 600 µl, was loaded onto the top of the gradient white slowly rotating the tube. The samples were centrifuged at 28 000 RPM for 3 h at 4°C using a Beckman SW28.1 rotor. After centrifugation, fractions were collected and measured using the Biocomp gradient station (30 fractions total). Fractions were flash-frozen and stored at –80°C before RNA extraction and library construction, and 20 µl of each fraction was saved for western blotting.

### Multiplex small RNA (MSR) sequencing of MCF7 tRNA and of input and polysome-associated mRNA

*tRNA:* MSR-seq was performed as previously described [37] with 1 µg total RNA input isolated from Q− or Q+ MCF7 cells using TRIzol extraction.

*mRNA:* MSR-seq can also be used to sequence poly(A)+ mRNA as described [37]. For mRNA sequencing, total RNA was extracted from input and polysome fractions using the TRIzol reagent (15596018, ThermoFisher), and MSR-seq libraries were built and analyzed as previously described [37]. Briefly, 3 volumes of TRIzol reagent were added to input lysate or pooled polysome fractions as indicated in Supplementary Fig. S4A for each sample. Total RNA was then extracted by following the manufacturer’s manual. Twenty micrograms of total RNA was used to extract poly(A)+ RNA using PolyATtract mRNA Isolation Systems (Z5310, Promega) by following the manufacturer’s manual. Four hundred nanograms of poly(A)+ RNA was fragmented at 94°C for 3 min (∼300 nt) using NEBNext Magnesium RNA Fragmentation Module (E6150S, NEB). Fragmented RNA mixture was then diluted to 50 µl using sterile H_2_O and purified using RNA Clean & Concentrator-5 (R1016, Zymo). RNA was eluted in 8 μl sterile H_2_O and 1 μl T4 PNK buffer and 1 µl 10 U/μl T4 PNK (EK0031, ThermoFisher) were added to each sample and incubated at 37°C for 30 min to repair the RNA ends, followed by inactivation at 75°C for 10 min. The end-repaired fragmented RNA (10 µl) was used as the input RNA for first adaptor ligation in MSR-seq. Standard MSR-seq procedure was followed afterwards. The PCR amplified MSR-seq libraries were purified using AMPure XP (A63880, Beckman Coulter) beads or gel.

### Western blots

Total protein extracts were obtained from MCF7 or HEK293T cells at ∼80% confluency. Protein lysate from each sample was boiled at 95°C for 5 min and loaded onto NuPAGE Bis-Tris protein gels and ran with 1× MES running buffer (ThermoFisher). Six pieces of MW blotting paper (MIDSCI), PVDF membrane (Millipore), gel, and six more pieces of MW blotting paper were layered in that order and placed into a Bio-Rad semi-dry transfer machine (Trans-Blot Turbo Transfer System). The membrane was blocked with 10% (w/v) blocking-grade buffer (Bio-Rad) in 1× TBST (National Diagnostics) overnight at 4°C. Washes were performed with 1× TBST. The membrane was washed 3 times for 5 min each and then incubated with a 5% (w/v) blocking-grade buffer in 1× TBST with the primary antibody at the manufacturer’s dilution or 1.5 h at room temperature. After 3 washes in 1× TBST, the membrane was incubated with the secondary antibody diluted in 1× TBST. One milliliter of Western-ECL substrate (Bio-Rad) was applied to the membrane and visualized with a ChemiDoc (Bio-Rad).

The antibodies used in this study are QTRT1 (Santa Cruz, sc-398918), QTRT2 (Santa Cruz, sc-377384), Vinculin (Santa Cruz, sc-73614), MSI2 (Abcam, ab76148), Lamin B1 (Abcam, ab16048), and α-Tubulin (Abcam, ab176560).

### Northern blots

Biotinylated probe for tRNA^His^ from IDT is displayed below:

/5Biosg/TGCCGTGACTCGGATTCGAACCGAGGTTGC TGCGGCCACAACGCAGAGTACTAACCACTATACGATCACGGC

Probes were radiolabeled with 5′ ^32^P by incubating 50 pmols of the probe with 1 µl 10× T4 PNK buffer, 2 µl 25 µM γ-³²P-ATP (150 µCi/µl), 5 µl ddH_2_O, and 1 µl T4 PNK (10 U/µl). The mix was incubated for 30 min at 37°C. Ten microliters of 2× RNA loading buffer was added and run on a 10% denaturing gel at 18 W. The bands were excised and placed into a 1.5 ml tube. The gel slices were crushed and covered in 900 µl of 200 mM KCl and 50 mM KOAc (crush-soak buffer) and rotated overnight at 4°C. The mix was centrifuged at 17 000 Gs for 1 min to pellet down agarose chunks. 2.7× of total volume of ethanol was added and the mix was put at −20°C for at least 1 h to precipitate the RNA. The tubes were centrifuged again at 4°C for 30 min and the supernatant was removed. Presence of radioactivity was measured with a Geiger counter. Resulting 5′ ^32^P-RNA was loaded onto a 10% denaturing gel and ran at 18 W for 1 h at 4°C. Transfer of the RNA was done with Hybond-XL membrane (GE Healthcare) under a vacuum gel dryer. Three layers of MW blotting paper (MIDSCI), membrane, and gel, in that order were incubated for 4 h at 80°C. The membrane was washed twice with 20 mM phosphate pH 7.0, 300 mM NaCl, 1% SDS (hybridization buffer) for 30 min each. The membrane was incubated with 5′ ^32^P DNA oligonucleotide probes in hybridization buffer for 16 h at 60°C in the UVP Hybridizer Oven. Membranes were washed three times for 20 min each in a 20 mM phosphate pH 7.2, 300 mM NaCl, 2 mM EDTA, and 1% SDS solution. The membrane was then scanned on a phosphor-imaging plate.

### High sensitivity northern blots for tRFs detection

Biotinylated probe for tRNA^Asn/Asp/Tyr/Lys/Gly/Glu^ from IDT have the following sequences:

tRNA^Asn^: /5Biosg/CGTCCCTGGGTGGGCTCGAACC ACCAACCTTTCGGTTAACAGCCGAACGCGCTAACCGATTGCGCCACAGAGAC

tRNA^Asp^: /5Biosg/CTCCCCGTCGGGGAATCGAACCCC GGTCTCCCGCGTGACAGGCGGGGATACTCACCACTATACTAACGAGGA

tRNA^Tyr^: /5Biosg/TCCTTCGAGCCGGASTCGAACCA GCGACCTAAGGATCTACAGTCCTCCGCTCTACCARCTGAGCTATCGAAGG

tRNA^Lys^: /5Biosg/CGCCCAACGTGGGGCTCGAACCCA CGACCCTGAGATTAAGAGTCTCATGCTCTACCGACTGAGCTAGCCGGGC

tRNA^Gly^: /5Biosg/TGCGTTGGCCGGGAATCGAACCCG GGTCAACTGCTTGGAAGGCAGCTATGCTCACCACTATACCACCAACGC

tRNA^Glu^: /5Biosg/TTCCCTGACCGGGAATCGAACCCG GGCCWMGGCGGTGARAGCGCCGAATCCTARCCACTAGACCACCAGGGR

Three μg of total RNA from each sample in 9 μl was mixed with 1 μl of Tris–HCl, pH 9, and incubated at 37°C for 30 min. Samples were then mixed with 10 µl of 2× RNA loading buffer and loaded onto 10% TBE–urea PAGE gel. The gel was run until the BPB dye came out. RNA was transferred to positively charged nylon membrane using Bio-Rad Trans-Blot Turbo Transfer System with 0.5× TBE for 30 min at constant 1A. The membrane was then crosslinked under UV three times with 120 mJ/cm^2^ each. The membrane was washed twice with 20 mM phosphate pH 7.0, 300 mM NaCl, 1% SDS (hybridization buffer) for 30 min each. Two hundred picomoles of 5′ biotinylated DNA probe in 25 µl was denatured at 95°C for 1 min, followed by immediate incubation on ice. The membrane was incubated with 200 pmol denatured probe in 50 ml of hybridization buffer for 16 h at 37°C. The membrane was washed twice with 20 mM phosphate pH 7.2, 300 mM NaCl, 2 mM EDTA, and 1% SDS buffer (wash buffer) at 37°C for 20 min each. The membrane was then incubated with streptavidin-HRP (Genscript, M00091, 1:5000 dilution) in 40 ml wash buffer at room temperature for 30–60 min, followed by three washes for 10 min each. The membrane was incubated with ultra-sensitive enhanced chemiluminescent (ECL) substrate (ThermoFisher, 34095) for 1 min and immediately imaged using Bio-Rad ChemiDoc MP imaging system.

### Msi2 knockdown and cell proliferation

siRNA 1 duplex was made with Millipore Sigma’s design tool:

Sense sequence: CCG AAU UGG GUU UAG UCA AUC[dT][dT]

Antisense sequence: GAUUGACUAAACCCAAUUCGG[dT][dT]

siRNA 2: Msi2 siRNA (h): sc-75834 (Santa Cruz Biotechnology, Inc.)

siRNA Ctrl: MISSION siRNA Universal Negative Control #1 (Millipore Sigma)

For each Msi2-siRNA duplex, 30 pmols were diluted in 500 µl of Opti-MEM. Five microliters of Lipofectamine 3000 was then added, and the mix was left at room temperature in each well of a six-well plate for 10 min. About 200 000 Q+ HEK293T cells were then plated into each well. Queuine was added to a final concentration of 1 µM for Q+ conditions. Cells were grown for 48 h and collected by incubation with 0.25% trypsin followed by centrifugation at 3000 rpm for 3 min. Cell pellets were resuspended in cell media containing dialyzed FBS. Approximately 50 000 cells were added with a multi-channel pipette to each well of a 96-well plate with, and queuine was again added to a final concentration of 1 µM for Q+ conditions. Ten microliters of CCK-8 solution was added to each well and plates were incubated for 2 h. Proliferation was measured with a microplate reader for the absorbance of 450 nm.

### Reverse transfection of tRF^His^

Sequences of RNA oligos from IDT used for tRF transfection:

tRF^His^: 5′-pGGCCGUGAUCGUAUAGUGGUUAGUACU CUGCGUU-3′

tRF^SC^: 5′-pGGUAGCGAAGCCUUUCGACGUUUGGUC UUGAUGU-3′

For each cell proliferation condition that included either tRF^His^ or tRF^SC^, 60 pmols of tRF oligo were mixed with 500 µl of Opti-MEM. Five microliters of Lipofectamine 3000 was added, and the mix was left at room temperature to incubate for 10 min. Subsequent cell proliferation experiments were carried out as described earlier.

### QTRT1 and QTRT2 shRNA knockdown and cell proliferation

Stable QTRT1 and QTRT2 shRNA knockdown and shRNA control MCF7 cell lines were performed as previously described [38]. Briefly, 1 × 10^5^ MCF7 cells/well were seeded into 12-well plate 24 h before lentiviral transduction. After 24 h incubation, the culture medium was replaced with complete medium containing 8 µg/ml polybrene. After thawing at room temperature, varying amounts of control, QTRT1, and QTRT2 shRNAs were added to each well at a multiplicity of infection of 2, 6, and 10. The cells were then incubated in CO_2_ incubator for 72 h, followed by treatment with 5 µg/ml puromycin. The cells were cultured with complete medium containing 5 µg/ml puromycin until all non-transduced cells were dead. QTRT1 and QTRT2 knockdown efficiency was examined using western blot before cell proliferation assay as mentioned above.

### tRF^His^ antisense oligo (ASO) knockdown and cell proliferation

**Table:** Sequences of ASOs from IDT used for tRF^His^ knockdown

Name	DNA sequence	IDT Affinity Plus gapmer-formatted
ASO-ctrl	GGCTACTACGCCGTCA	+G*+G*+C*T*A*C*T*A*C*G*C*C*G*+T*+C*+A
ASO-1	ACTATACGATCACGGC	+A*+C*+T*A*T*A*C*G*A*T*C*A*C*+G*+G*+C
ASO-2	CCACTATACGATCACG	+C*+C*+A*C*T*A*T*A*C*G*A*T*C*+A*+C*+G

ASOs were reverse transfected into Q− MCF7 cells. Briefly, Q− MCF7 cells were cultured to 80% before collection and counting. At the same time, ASO complex was prepared for each ASO, 240 pmols ASO were diluted in 500 µl of Opti-MEM in six-well plate with gentle agitation. Five microliters of lipofectamine RNAiMAX was then added to each well and mixed with gentle agitation. The ASO complex was incubated at room temperature for 15 min. 6 × 10^5^ Q− MCF7 cells were then added to each well and mixed with gentle agitation. Complete medium was added to each well to 3 ml and the cells were incubated with ASO for 72 h before cell proliferation assay or northern blots.

For cell proliferation assay, after incubation, ASO-treated MCF7 cells were collected and counted. At the same time, ASO transfection mixture for one well of six-well plate was prepared as above for double ASO transfection to keep tRF^His^ knockdown efficiency over the course of proliferation assay. For each ASO treated condition, 6 × 10^5^ cells were added to each 15-ml conical tube. The prepared ASO mixture was then added to each corresponding 15-ml conical tube, followed by the addition of complete medium to 6 ml. Each cell mixture was then mixed and seeded into 6 × 96-well plates (one column per plate and 100 μl cells per well) using multi-channel pipet for 6-day proliferation assay. At the same time of each day, 10 µl CCK-8 reagents were added to each well and incubated for 2 h before measurement of absorbance at 450 nm using microplate reader.

For northern blots, after incubation, medium was completely removed from each well (triplicates for each ASO), followed by 1× wash with cold 1× PBS. Five hundred microliters of TRIzol Reagent was added directly to each well followed by incubation at room temperature with shaking at 210 RPM for 15 min. The TRIzol mixture from each well was then transferred to 1.5-ml microcentrifuge tubes. Total RNA was extracted by following manufacturer’s manual. High-sensitivity northern blots were performed as above to check tRF^His^ knockdown efficiency.

## Results

### Increased tRNA queuosine modification reduces proliferation of MCF7 cells, but can be rescued by a tRNA fragment

To investigate Q-dependent effects on proliferation, we grew multiple cell lines in a queuine-devoid environment (Q−) by using dialyzed FBS in our cell media; conversely, 1 µM queuine was added to generate “Q+” cells. The Q-modification levels in Q− and Q+ cells were confirmed using northern blot probes with APB gels [21] (Supplementary Fig. S1B). We tested six lines of Q− and Q+ cells for proliferation. They include human cervical cancer cells (HeLa), mouse embryonic fibroblasts (MEFs), non-tumorigenic breast epithelial cells (MCF10A), ER− breast cancer cells (MDA-MB-231), human colorectal carcinoma cells (HCT116), and ER+ breast cancer cells (MCF7) (Fig. 1A and Supplementary Fig. S1C). No differences in proliferation in 4 of the 6 cell lines were observed, while MCF10A showed increased proliferation, and MCF7 showed decreased proliferation in the Q+ condition. In a separate study from our laboratory, we showed that queuine increases proliferation of HEK293T (transformed embryonic kidney cells) and BMDC (primary mouse bone marrow dendritic cells) [34]. To associate queuine-dependent effect on MCF7 proliferation to tRNA Q-modifications, we knocked down QTRT1 or QTRT2 (Fig. 1B), subunits of the Q-modification writer complex, and found that reducing either protein level increased proliferation of MCF7 Q+ cells (Fig. 1C and Supplementary Fig. S1D). Unexpectedly, Q− cell proliferation also increased upon QTRT1 or QTRT2 knockdown (Supplementary Fig. S1D), suggesting that QTRT1/QTRT2 may play an uncharacterized role in addition to tRNA modification in MCF7 proliferation.

**Figure 1.**
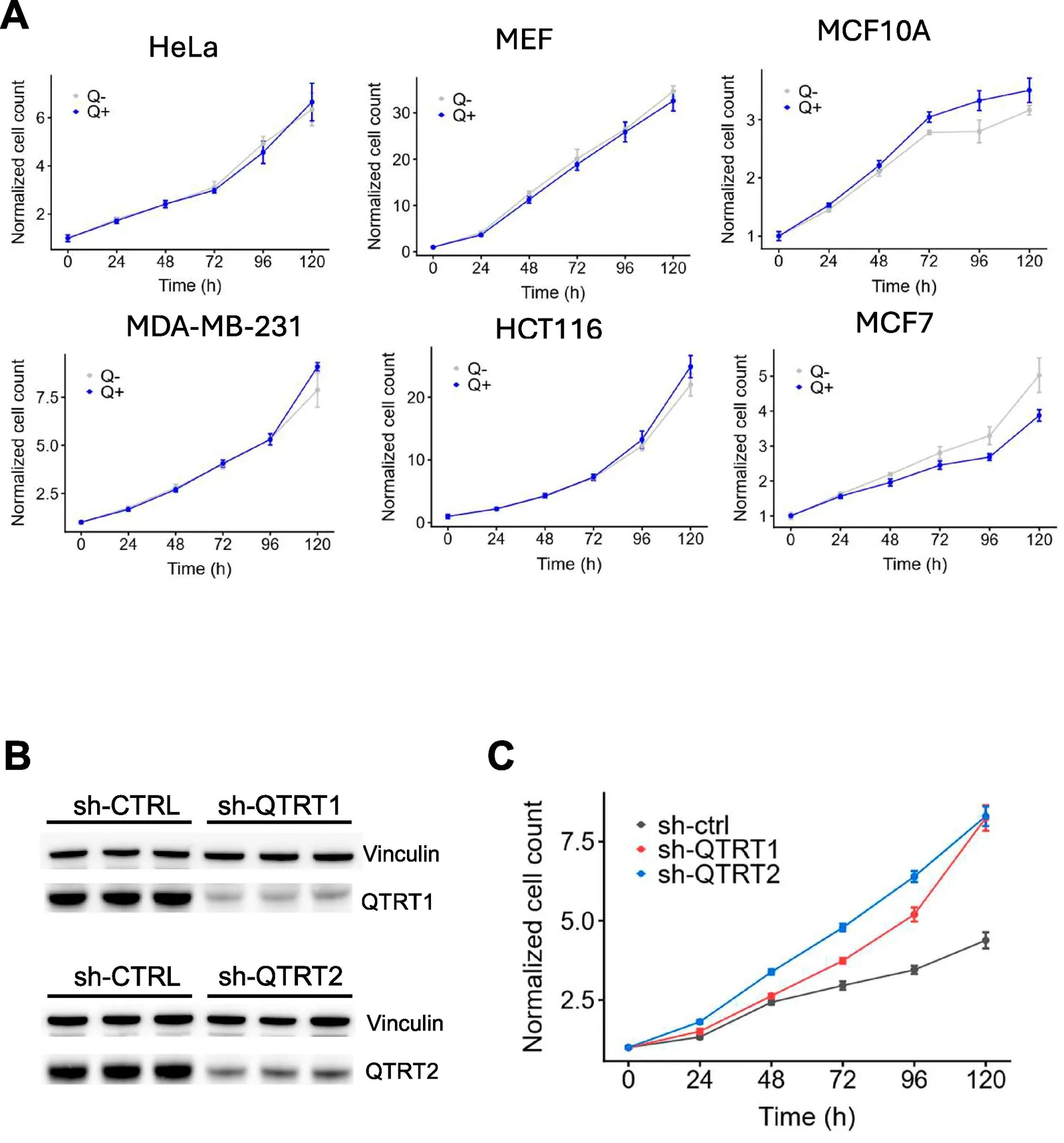
Effect of queuosine modification on cell proliferation is cell type and QTRT1/QTRT2 dependent. (**A**) Proliferation curves of HeLa, MEF, HCT116, MCF10A, MDA-MB-231, and MCF7 without (Q−) or with 1 µM queuine (Q+) addition. (**B**) Western blot validation of QTRT1 and QTRT2 stable knockdown using shRNAs, shown in biological triplicates. Vinculin is the loading control. (**C**) Proliferation curve of MCF7 cell proliferation after QTRT1 and QTRT2 stable knockdown using shRNAs under Q+ condition.

MCF7 cells highly express both ER-alpha and ER-beta receptors [39]. Estrogen receptors (ERs) and ER-containing cell lines have been a focus of study, as high expression of these hormone receptor proteins are associated with increased risk of breast cancer, and over 50% of all breast cancers express ERɑ [39–41]. A previous study showed that specific tRNA fragments such as tRF^His^ were expressed in ER+ breast cancer cells, including MCF7, and in AR+ prostate cancer cells [29]. tRNA^His^ is a Q-modifiable tRNA. tRNA Q-modification is known to affect tRF biogenesis [21]. We measured tRF^His^ levels in Q− and Q+ MCF7 cells by high-sensitivity northern blots and found significantly higher levels of two tRF^His^ bands in Q− cells (Fig. 2A), as well as increased tRF^Asn^ level derived from the Q-modifiable tRNA^Asn^, but not statiscally significant difference in the levels of tRF^Asp^ or tRF^Tyr^ and several non-Q-modifiable tRNAs (Supplementary Fig. S2A). We carried out multiplex small RNA sequencing (MSR-seq, [37]) for Q− cells that can simultaneously access full-length tRNA and tRNA fragments (Fig. 2B). We found two prominent 5′ tRNA fragments that approximately correspond to band 1 and 2 in the Northern blot, one with the 3′ end in the anticodon loop, the other in the variable loop.

**Figure 2.**
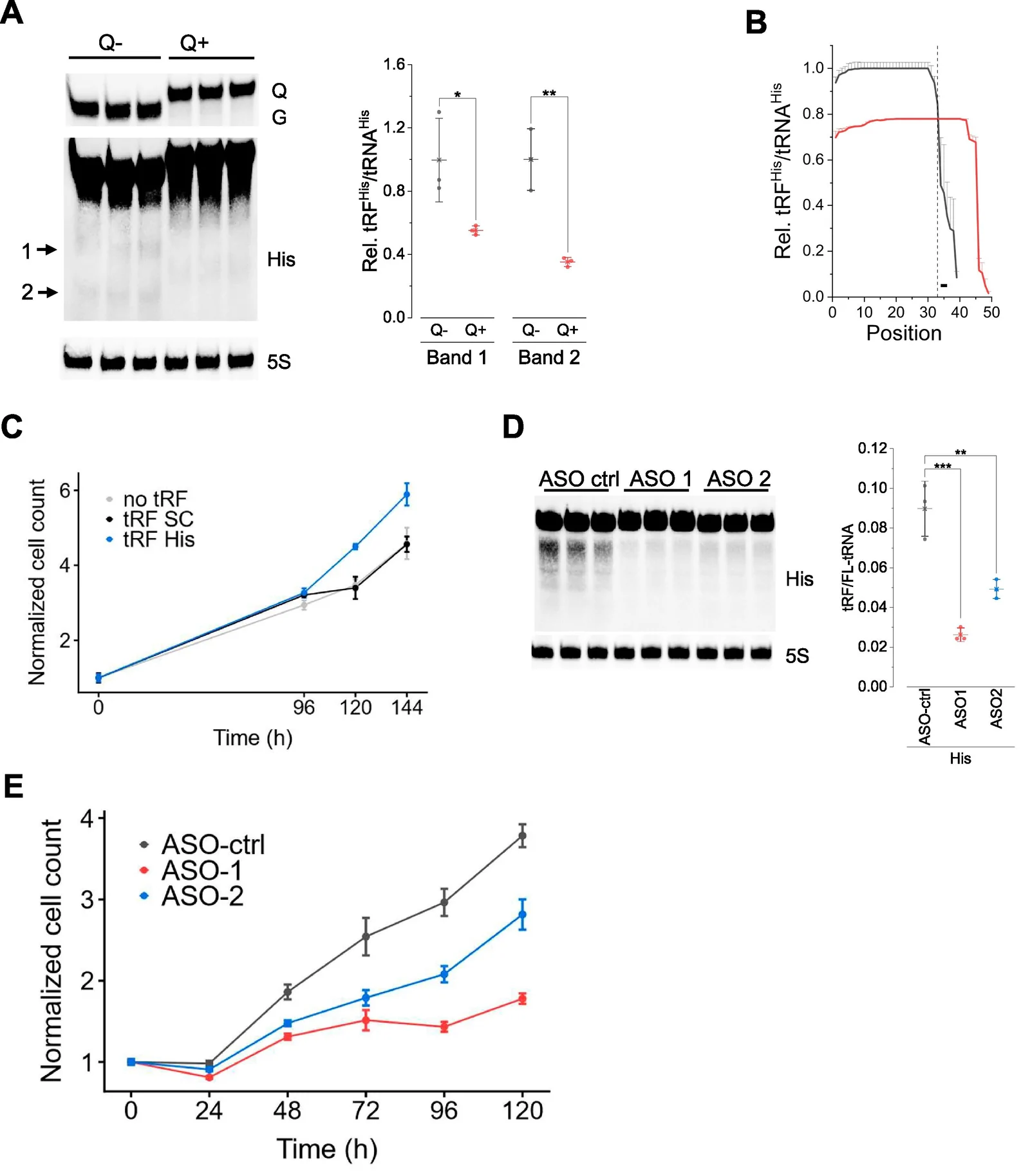
Q-modification affects tRF^His^ generation, and tRF^His^ promotes MCF7 proliferation. ns: not significant, *: *P* < .05, **: *P* < .01, ***: *P* < .001. (**A**) Northern blot result of tRF^His^ and full-length tRNA^His^ in Q− and Q+ MCF7 cells, 5S RNA is the loading control. Left: northern blot. Right: quantification of the relative levels of tRF^His^ bands. (**B**) tRF^His^ result from MSR-seq [39] that simultaneously measures full-length tRNA and tRNA fragments. *n *= 3 biological replicates. Anticodon nucleotides 34–36 are indicated by a thick bar. The tRF^His^ oligo used for cell proliferation study has a 3′ end indicated by a dashed line. Q− cells were obtained through multiple passages and cell growth in dialyzed FBS. To obtain Q+ cells, 1 µM of queuine was added to Q− cell culture and grown for additional 24 h. (**C**) Proliferation curves of MCF7 cells upon tRF^His^ or scrambled control tRF^SC^ transfection of Q+ cells. (**D**) Northern blots of Q-modified tRNAs upon tRF^His^ knockdown by antisense oligos (ASO). 5S is the loading control and quantitation relative to full-length tRNA^His^ (right). (**E**) Proliferation curves of MCF7 cell proliferation upon transfection of control or tRF^His^-targeting antisense oligos in Q− cells.

We then asked if Q-modification-dependent tRF^His^ is responsible for the proliferative changes in MCF7 cells. We selected one specific tRF^His^ with the 3′ end at position 33 corresponding to band 2. Indeed, we saw a significant increase in cell proliferation upon transfecting a tRF^His^ oligonucleotide into Q+ MCF7 cells compared to the control transfection of a scrambled tRF^SC^ (Fig. 2C and Supplementary Fig. S2B). This effect remained the same when varying the transfection amount of tRF^His^/tRF^SC^ over a 9-fold range (Supplementary Fig. S2C and D), indicating that the proliferative effect is reproducible across multiple input doses. We note that the nominal amount of transfected tRF cannot be directly compared with endogenous tRF^His^ levels, because transfected RNA is inefficiently released from endosomes, and only a minor fraction is expected to become bioavailable in the cytosol [42–45]. Therefore, while the transfection experiment does not precisely define the physiological cytosolic concentration of tRF^His^, the dose-range analysis suggests that the growth-promoting effect is not dependent on a single excessive input concentration. Furthermore, we used antisense oligonucleotides to specifically knock down the endogenous tRF^His^ in Q− MCF7 cells, while the tRF levels of the 3 other Q-modifiable tRNAs or non-Q-modified tRNAs remained unchanged (Fig. 2D and Supplementary Fig. S2E). Knocking down the endogenous tRF^His^ significantly reduced proliferation of MCF7 Q− cells (Fig. 2E and Supplementary Fig. S2F). Unexpectedly, tRF^His^ knockdown also reduced proliferation of Q+ cells (Supplementary Fig. S2G), suggesting that the tRF knockdown strategy may be inadequate to assess cell proliferation. In any case, these results indicate that tRF^His^ levels affect the proliferation of MCF7 cells.

### tRNA Q-modification moderately affects other translation-promoting tRNA modifications

To analyze the molecular basis of the Q-dependent proliferation in MCF7 cells, we carried out multiplex small RNA sequencing [37], which examines tRNA abundance and modification, on Q− and Q+ cells. As expected, the tRNA^His^ Q-modification levels are significantly different in Q− and Q+ cells as determined by the sequencing signatures of tRNA^His^ in our PAQS-seq data analysis procedure ([46], Supplementary Fig. S3A). At the anticodon family level, no significant change of >1.5-fold in tRNA abundance was present (Fig. 3A). In both Q− and Q+ cells, the abundance of the 4 Q-modified cytosolic tRNAs differed by ∼20-fold, with tRNA^Asp^ at the highest and tRNA^His^ at the lowest level, even though the genomic codon usage among these 4 amino acids differs only by ∼1.9-fold. Similarly, the abundance of mitochondrial Q-modified tRNAs differ by ∼10-fold, but the codon usage differs by ∼2.5-fold (Fig. 3B and Supplementary Fig. S3B). These results do not obviously implicate tRNA abundance as a driver for Q-dependent differences in MCF7 proliferation.

**Figure 3.**
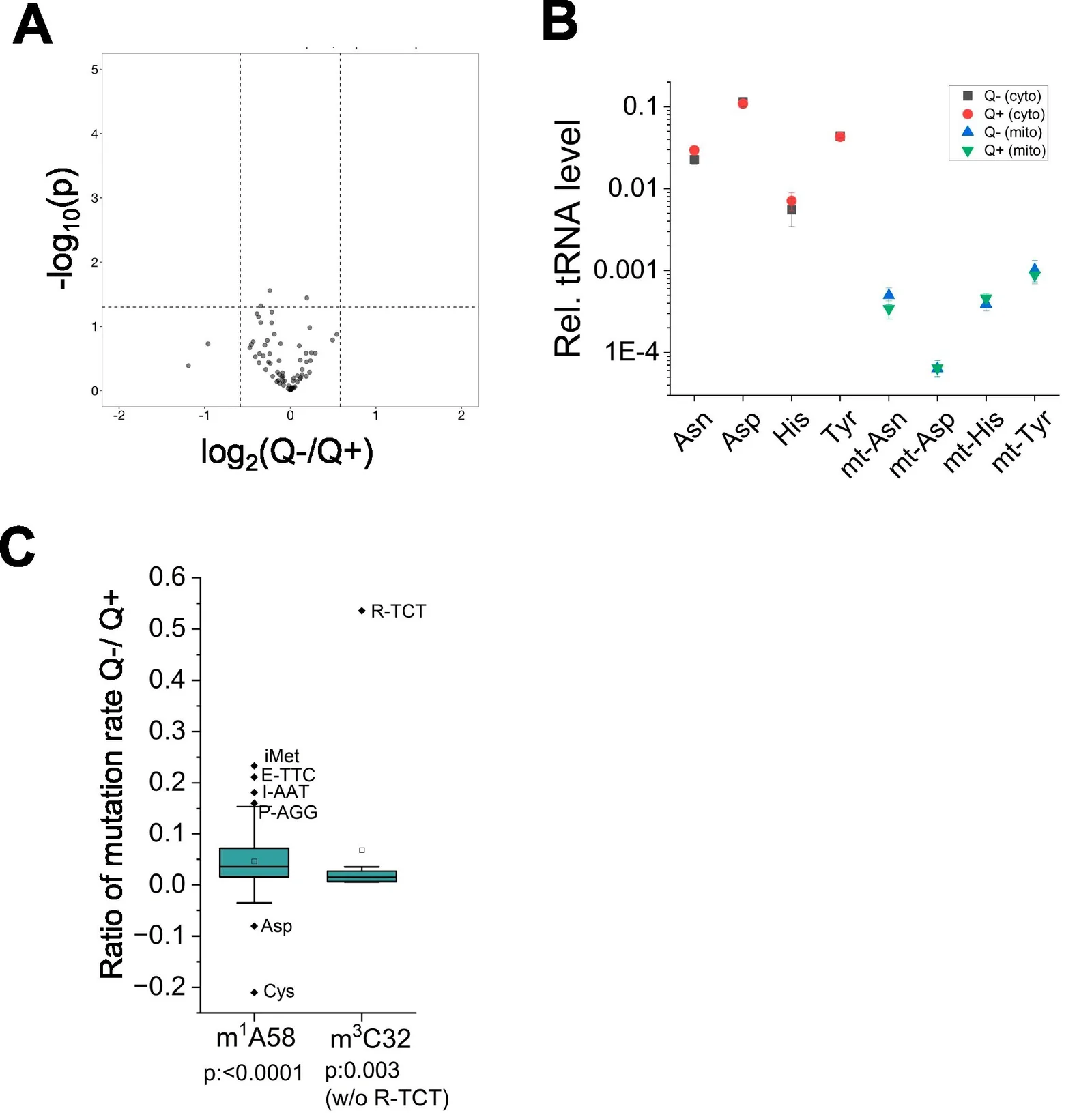
Queuine treatment of MCF7 cells does not change tRNA abundance but alters additional tRNA modification. (**A**) Volcano plot of tRNA-seq data of full-length tRNA abundance in Q− and Q+ cells shows no significant changes in tRNA abundance within 1.5-fold. (**B**) Q-modified cytosolic and mitochondrial tRNA abundance in Q− and Q+ cells. (**C**) m^1^A58 and m^3^C32 changes in Q− and Q+ cells measured by the differential mutational rate in sequencing. Only the most abundant isodecoder in each tRNA anticodon family and the average mutation rate among three biological replicates are shown here. Data points *n *= 46 for m^1^A58, *n *= 9 for m^3^C32. Outliers are labeled with tRNA names, including anticodon for isoacceptors.

We also compared the mutation rates of Q− and Q+ cells in MSR-seq for the two translation-promoting modifications, m^1^A58 and m^3^C32 [47–49] (Fig. 3C). Both modifications are methylations at the base-pairing face that disallow standard base pairing during cDNA synthesis by the reverse transcriptase. When using a processive RT in the MSR-seq procedure [37], the RT can read through these modified nucleotides, leaving behind a mutation signature in the sequencing reads. We have shown previously that the mutation fraction for m^1^A and for m^3^C can be used to semi-quantify modification levels [37, 50]. m^1^A58 occurs in the T loop in all human cytosolic tRNAs, its positive charge enhances tRNA-ribosome interactions. m^3^C32 occurs in the anticodon loop in tRNA^Arg^ (CCT), tRNA^Arg^ (TCT), tRNA^Ser^, and tRNA^Thr^; this methylation strengthens the anticodon-codon interactions. At the overall level, we found ∼5% increase of m^1^A58 in Q− cells. m^3^C32 showed a ∼2% increase for tRNA^Arg^ (CCT), tRNA^Ser^, and tRNA^Thr^; however, tRNA^Arg^ (TCT) seems to be an outlier with a much larger increase. This result may be related to the formation of a QTRT2-METTL2A (tRNA^Arg^ (TCT) m^3^C writer) complex identified in a proteome-wide study [51]. Increased methylation levels at these locations may moderately support an increase in translation efficiency in Q− cells.

### tRF^His^ increases translation in MCF7 cells

To investigate how tRFs might impact translation (e.g. [22, 52]) in MCF7 cells, we performed polysome profiling of Q+ MCF7 cells that were transfected with tRF^His^ or tRF^SC^ control and found that tRF^His^ transfection increased the polysome density (Supplementary Fig. S4A). Among the mRNA transcripts, we found very little difference in the input samples between tRF^His^ and tRF^SC^ transfected cells (Fig. 4A, left). On the other hand, substantial differences were found in the polysome associated mRNAs (Fig. 4A, right). Comparing translation efficiency (TE), which corresponds to the individual mRNA transcript of polysome/input samples, we identified 395 gene transcripts that were upregulated, and 303 transcripts that were downregulated in tRF^His^ over tRF^SC^ transfected cells (Fig. 4B and Supplementary Fig. S4B). Gene Ontology (GO) analysis of the upregulated genes showed enrichment for genes involved in cell metabolism, while the downregulated genes showed enrichment in signal transduction (Fig. 4C). This result suggests that tRF^His^ transfected cells translate more proteins in cellular metabolic and biosynthetic processes, which likely increases the cellular metabolic activity.

**Figure 4.**
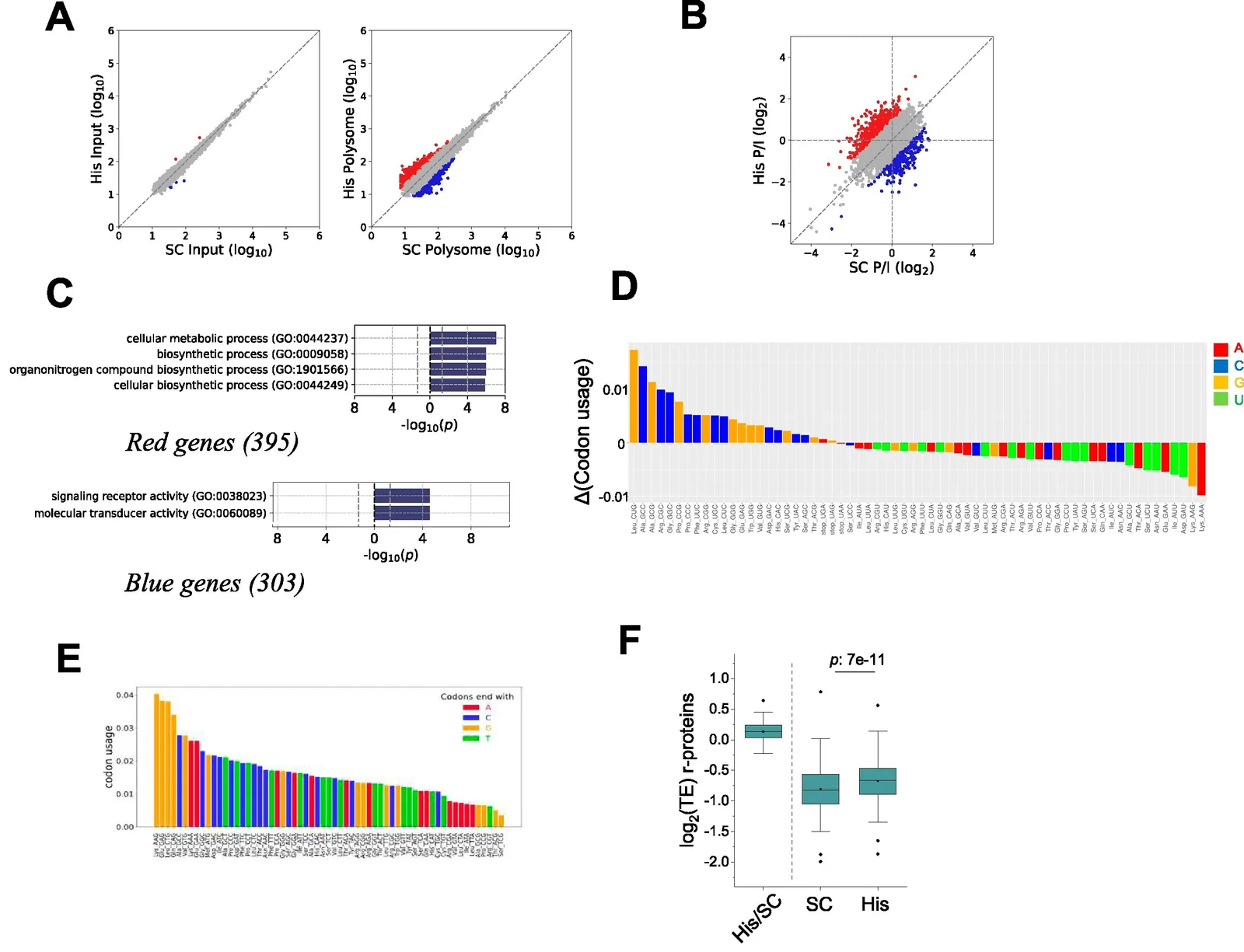
tRF^His^ effect on MCF7 cell proliferation is mostly on translation. (**A**) mRNA level differences of input (left) and on polysome (right) of tRF^His^ and tRF^SC^-transfected cells. (**B**) Translation efficiency (TE) between tRF^His^ and tRF^SC^-transfected cells. (**C**) GO of genes with higher [left, red genes in panel (B)] or lower [right, blue genes in panel (B)] TE in tRF^His^ over tRF^SC^-transfected cells. (**D**) Codon usage of red gene over blue genes shows a preference for C/G-ending codons in up-regulated genes. (**E**) Codon usage of all codons of human ribosomal protein genes. Codons are color-coded by third base. (**F**) TE of ribosomal protein genes in tRF^His^ over tRF^SC^-transfected cells.

Comparing the codon usage of the upregulated versus the downregulated group of genes on the polysome, we found a significant preference of C/G-ending codons in the upregulated genes (Fig. 4D). One major group of house-keeping genes whose high level of translation is required for cell proliferation includes ribosomal proteins [53, 54]. Human ribosomal protein genes also significantly favor C-ending over U-ending codons (Fig. 4E). Focusing on the ribosomal protein genes as a group, we found that their translation efficiency in tRF^His^-transfected MCF7 cells is significantly higher than that in tRF^SC^-transfected cells (Fig. 4F). The C/G-ending preference of transcripts with a high TE revealed by polysome profiling favors the translation of both metabolic enzymes and ribosomal proteins, which can account for the increased rate of proliferation upon tRF^His^ transfection.

We also found differences in the translation efficiency of tRNA modification enzymes (Supplementary Fig. S4C). Translation decreased for the Q-writer, QTRT1, upon tRF^His^ transfection, suggesting that MCF7 cells may aim to retain a low Q-modification state during proliferation. Since we used 1 µM queuine in our study, this decrease may not be sufficient to reduce Q-modification levels. On the other hand, under the same condition, translation increased for the m^1^A writer, TRMT61A, and did not significantly change for the m^3^C writer, METTL2A. The increased tRNA m^1^A or m^3^C levels (Fig. 3C and D) may be derived from other cellular activities such as post-translational modification of the m^1^A or m^3^C writer.

### Msi2 protein binds tRF^His^

Since tRFs are known to bind specific cellular proteins to impart function [26, 52], we adopted a biochemical approach to identify tRF^His^ binding proteins. Since the tRF^His^ effect on cell proliferation is on translation, we reasoned that the relevant tRF^His^ interacting proteins should be located in the cytoplasm. We first showed that the native tRF^His^ is indeed present at appreciable levels in the cytoplasm using cellular fractionation of MCF7 cells and northern blot (Fig. 5A and Supplementary Fig. S5A and B). We therefore used immobilized tRF^His^ as the bait to pull down cytoplasmic proteins, while using immobilized tRF^SC^ as a control (Fig. 5B). Indeed, substantial differences in pull-down proteins were found, where three main protein band groups can be seen at ∼55, ∼38, and ∼32 kDa (Fig. 5C). We then performed MS analysis of these proteins, focusing on those between 30 and 40 kDa (Fig. 5D). Among the top 10 proteins, 6 were hnRNP proteins that are known to mostly bind mRNAs [55, 56], and 3 were ribosomal or ribosome maturation proteins. Excitingly, we found the Musashi RNA binding protein 2 (Msi2) among the top 10 proteins (Fig. 5E). Msi2 has been shown to bind tRFs in a previous study [57] and has been shown to be a regulator of proliferation in pancreatic beta cells through increased mitochondrial activity. Consistent with this idea, our mRNA-seq result shows increased levels of mt-mRNA coding for complexes III and IV (Supplementary Fig. S5C).

**Figure 5.**
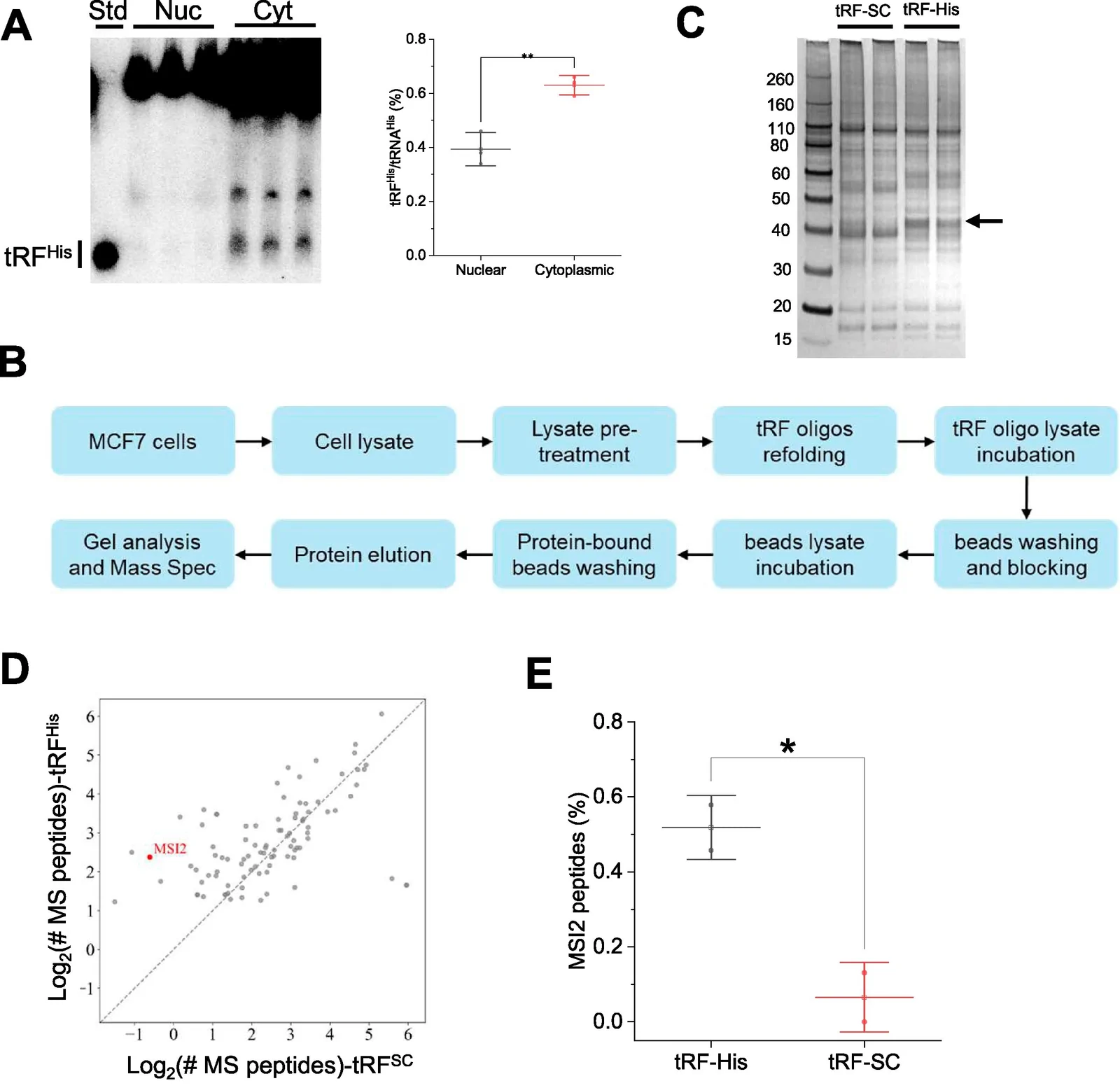
tRF^His^ oligonucleotide preferentially pulls down Msi2 protein. ns: not significant, *: *P* < .05, **: *P* < .01. (**A**) Northern blot of tRF^His^ in MCF7 nuclear (Nuc) and cytoplasmic (Cyt) fractions together with tRF^His^ standard (Std). Right: quantification of tRF^His^ abundance relative to full-length tRNA^His^. (**B**) Workflow of the tRF^His^/tRF^SC^ pull-down and protein mass spectrometry experiment. (**C**) Analysis of tRF^His^ or tRF^SC^ pull-down proteins using MCF7 cytoplasmic fraction shown by SDS–PAGE and silver stain. Arrow points to the protein band containing the MSI2 protein. (**D**) Scatter plot of top 100 proteins in the pull-down protein mass spectrometry data, tRF^His^ versus tRF^SC^. The MSI2 protein is highlighted in red. *n *= 2 biological replicates. (**E**) Relative abundance of the MSI2 protein in tRF^His^ and tRF^SC^ pull-down samples.

### Q modification effect on MCF7 proliferation is Msi2-dependent

To investigate whether Msi2 interaction with tRF^His^ plays a role in MCF7 proliferation, we first measured cell proliferation in Q− and Q+ cells upon knocking down the Msi2 protein (Fig. 6A and Supplementary Fig. S6A). Knocking down Msi2 significantly reduced cell proliferation for Q− cells but had little effect on proliferation of Q+ cells (Fig. 6B and Supplementary Fig. S6B). To further associate Msi2 protein effect with tRF^His^, we carried out a combined si-Msi2 and tRF transfection and cell proliferation experiment (Fig. 6C). We only used Q+ cells here because of their low levels of endogenous tRF^His^ and low sensitivity to si-Msi2 knockdown. First, comparing si-CTRL cells showed that tRF^His^ transfection significantly increased proliferation compared to tRF^SC^ as expected (Fig. 6D and Supplementary Fig. S6C). Among the two si-Msi2 RNAs used, tRF^His^ no longer increased MCF7 proliferation compared to tRF^SC^ for siRNA1 (Fig. 6D). Even though tRF^His^ still increased proliferation for siRNA2 over tRF^SC^, the magnitude of increase was decreased compared to si-CTRL (Fig. 6D). This result can be better visualized by plotting the relative MCF7 proliferation ratio of tRF^His^/tRF^SC^ upon si-Msi2 knockdown (Fig. 6E). Focusing on the 72 h time point, si-CTRL showed increased proliferation for tRF^His^ over tRF^SC^, whereas si-Msi2 reduced the tRF^His^ over tRF^SC^ effect compared to si-CTRL. Together, these results indicate that the Q-modification and tRF^His^ effect on MCF7 cell proliferation is dependent on the Msi2 protein.

**Figure 6.**
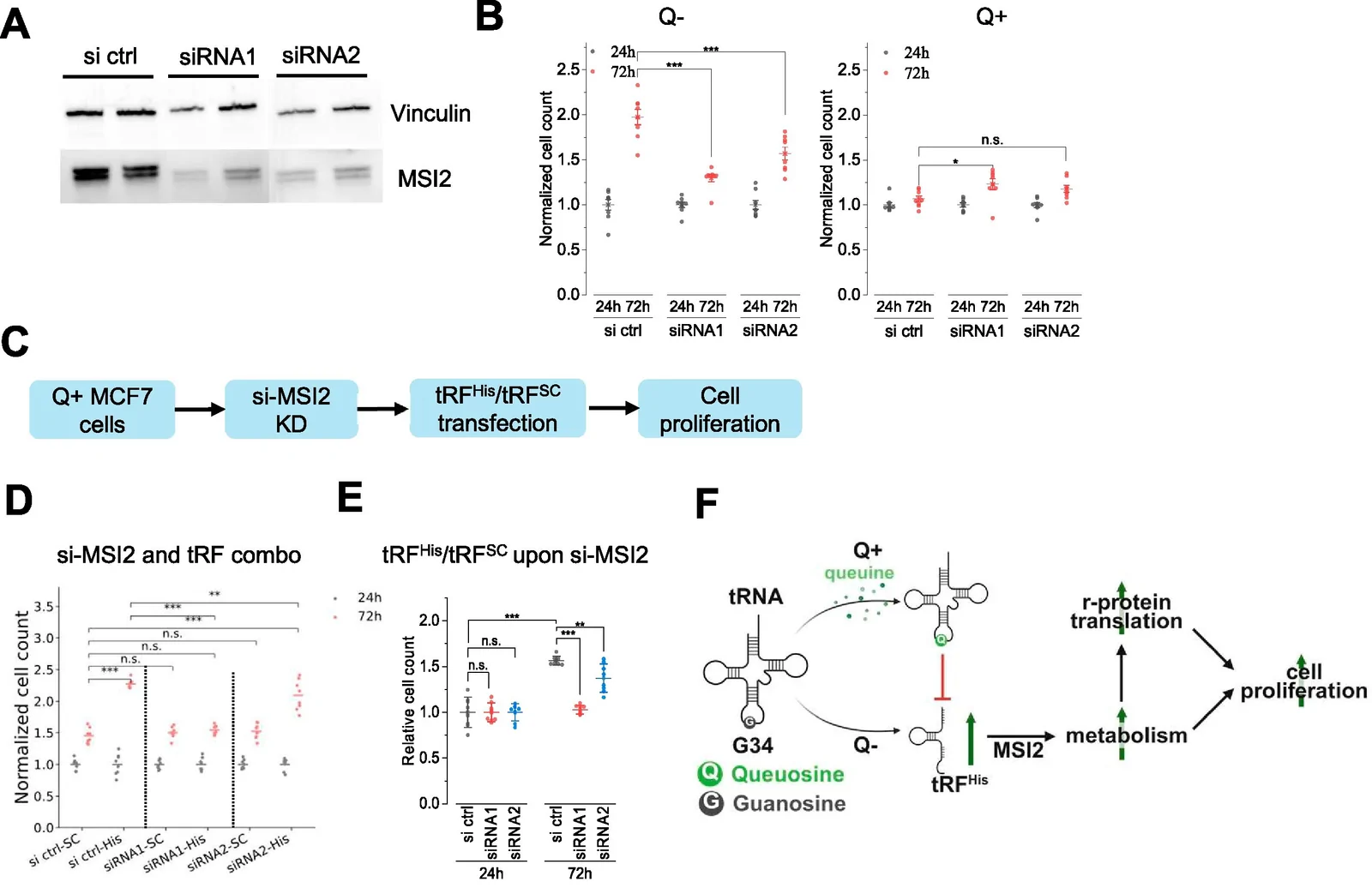
Msi2 protein facilitates the tRF^His^ promotion of MCF7 cell proliferation. ns: not significant, *: *P* < .05, **: *P* < .01, ***: *P* < .001. (**A**) Western blot validation of Msi2 protein knockdown. Vinculin is the loading control. (**B**) Proliferation of Q− (left) or Q+ (right) MCF7 cells upon MSI knockdown. (**C**) Experimental workflow of si-MSI2 knockdown and tRF^His^/tRF^SC^ transfection in Q+ MCF7 cells. (**D**) Proliferation of Q+ MCF7 cells with si-MSI2 knockdown and tRF^His^ or tRF^SC^ combination, comparing individual conditions. Dashed lines separate the si-CTRL, si-Msi2-1, and si-Msi2-2 data points. (**E**) Proliferation of Q+ MCF7 cells with si-MSI2 knockdown and tRF^His^ or tRF^SC^ combination, comparing tRF^His^/tRF^SC^ upon siMSI2 knockdown. (**F**) Model of queuine and tRF^His^ dependent MCF7 cell proliferation. Depletion of Q-modification increases tRF^His^ levels, its interaction with Msi2 protein increases cellular metabolism [57], which increases ribosomal protein translation and proliferation.

We summarize our findings here in a model for queuine and tRF^His^ dependent MCF7 cell proliferation (Fig. 6F). Decreasing Q-modification increases tRF^His^ levels. tRF^His^ interacts with the Msi2 protein, which increases cellular metabolism [55]. This results in increased translation of ribosomal proteins that speeds up cell proliferation.

## Discussion

Previous studies have demonstrated that the angiogenin ribonuclease is responsible for cleaving mature tRNAs at the anticodon loop [29, 57–59] and that the accumulation of these tRFs are functionally involved in cell proliferation [29, 57]. In ER+ breast and AR+ prostate cancer cell lines, tRF halves are constitutively generated and are dependent on the presence of the sex-dependent hormones [29]. Since tRNA Q-modification is known to protect tRF biogenesis [21], we set out to associate tRNA Q-modification and tRFs, from Q-modified tRNAs, with cell type-dependent proliferation. Consistent with the previous report [29], we found that the Q-modification dependent tRF^His^ is sufficient to increase proliferation of ER+ breast cancer MCF7 cells, most likely through its interaction with the Msi2 protein. MCF7 cells lacking tRNA Q-modification are also accompanied by increased levels of m^1^A and m^3^C modifications in other cellular tRNAs that may also promote translation efficiency, although the mechanism of this connection is still unclear. Transfection of tRF^His^ versus tRF^SC^ control promotes translation of gene transcripts enriched in C/G-ending codons, including those of ribosomal proteins, an essential housekeeping gene group required for rapid cell proliferation [53, 54].

Both Msi1 and Msi2 are known RNA-binding proteins that regulate the translation of gene transcripts in key oncogenic pathways and have been linked to various cancer types [40]. A previous study showed that both proteins can bind tRFs and elucidated Msi1’s role in the localization of tRFs to the mitochondria, contributing to neonatal beta cell proliferation [57]. In this study, we find that Msi2 is a key binding protein to tRF^His^, which mediates tRNA Q-modification-dependent proliferation effect in MCF7 cells. Further work will investigate why tRF^His^ and Msi2 interaction specifically affects cell proliferation in ER+ breast cancer.

In summary, we find that tRNA Q-modification has a strong cell-context dependence on mammalian cell proliferation. Only two of the six cell lines examined here showed significant proliferation differences, and one showed unanticipated slower proliferation at higher tRNA Q-modification levels. This cell context dependence points to the complexity of Q-modification effects that need to be studied on a cell-type-dependent basis.

## Supplementary Material

gkag1101_Supplemental_File

## Data Availability

The mRNA and tRNA seq data are deposited to NCBI GEO under the accession numbers: GSE293473 and GSE293476, respectively.
