# Supplementary material for: Queuosine-modification dependent histidyl tRNA fragment promotes cell-type dependent proliferation
Source: Nucleic Acids Res. 2026 Oct 31;54(20):gkag1101. doi: 10.1093/nar/gkag1101 (PMC13722870; doi:10.1093/nar/gkag1101)
Supplement: Supplementary file 1 [file gkag1101_supplemental_file.pdf]

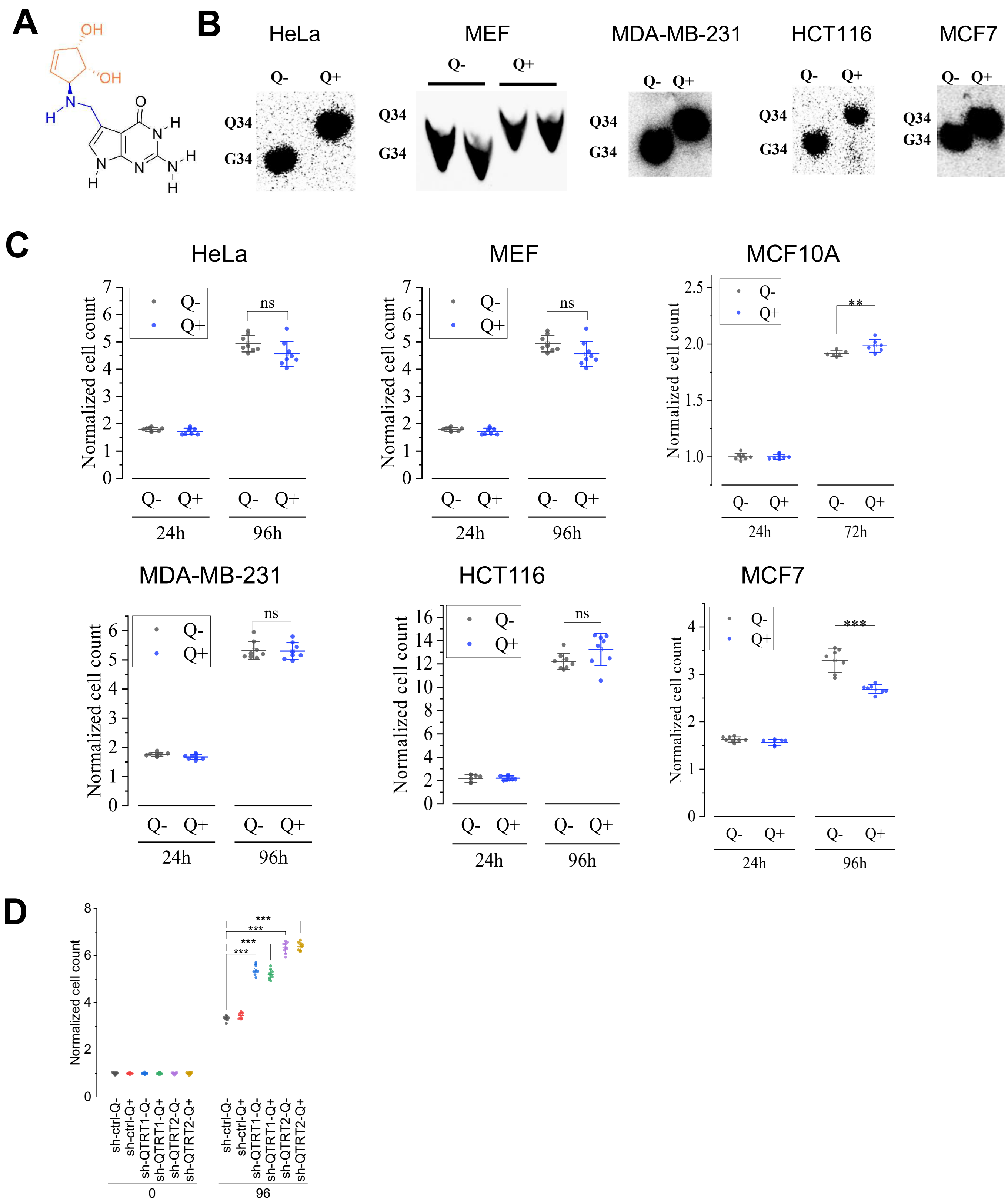

Figure S1. Additional data for Figure 1.

(A) Structure of queuosine.

(B) Northern blot validation of Q- and Q+ status in HeLa, MEF, HCT116, MDA-MB-231, and MCF7 using APB gel, tRNA^{Asn} probe.

(C) Proliferation of HeLa, MEF, HCT116, MCF10A, MDA-MB-231, and MCF7 without (Q-) or with 1 μM queuine (Q+) addition.

(D) MCF7 cell proliferation after QTRT1 and QTRT2 stable knockdown using shRNAs without (Q-) or with 1 μM queuine (Q+) addition.

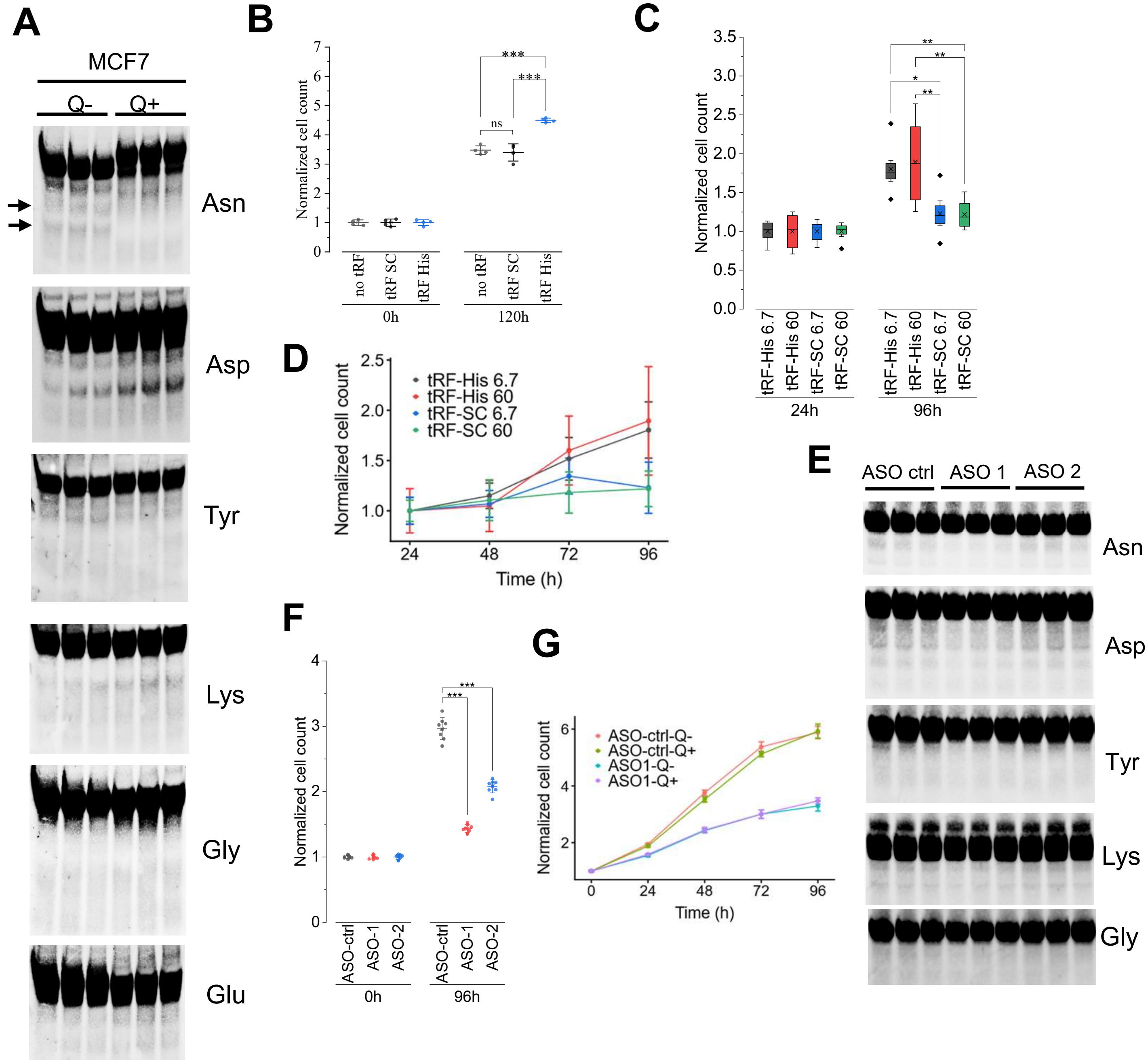

Figure S2. Additional data for Figure 2. *: $p<0.05$, **: $p<0.01$, ***: $p<10^{-3}$, *ns*: not significant.

(A) Northern blot of other Q-modified tRNAs (Asn, Asp, Tyr) and not-Q-modified tRNAs (Lys, Gly, Glu) for tRF detection. $tRF^{Asp}/tRNA^{Asp}$ differences are not statistically significant.

(B) Proliferation of MCF7 cells upon tRF^{His} or scrambled control tRF^{SC} transfection of Q+ cells.

(C) MCF7 cell proliferation after 6.7pmol or 60pmol/10-cm plate tRF^{His} or scrambled control tRF^{SC} transfection of Q+ cells.

(D) Proliferation curves of MCF7 cells after 6.7pmol or 60pmol/10-cm plate tRF^{His} or scrambled control tRF^{SC} transfection of Q+ cells.

(E) Northern blots of other Q-modifiable tRNAs (Asn, Tyr, Asp) and not-Q-modifiable tRNAs (Lys, Gly) upon tRF^{His} knockdown by antisense oligos (ASO).

(F) MCF7 cell proliferation after transfection of control or target antisense oligos of tRF^{His} of Q- cells.

(G) MCF7 cell proliferation after transfection of control or target antisense oligos of tRF^{His} of Q- and Q+ cells.

A

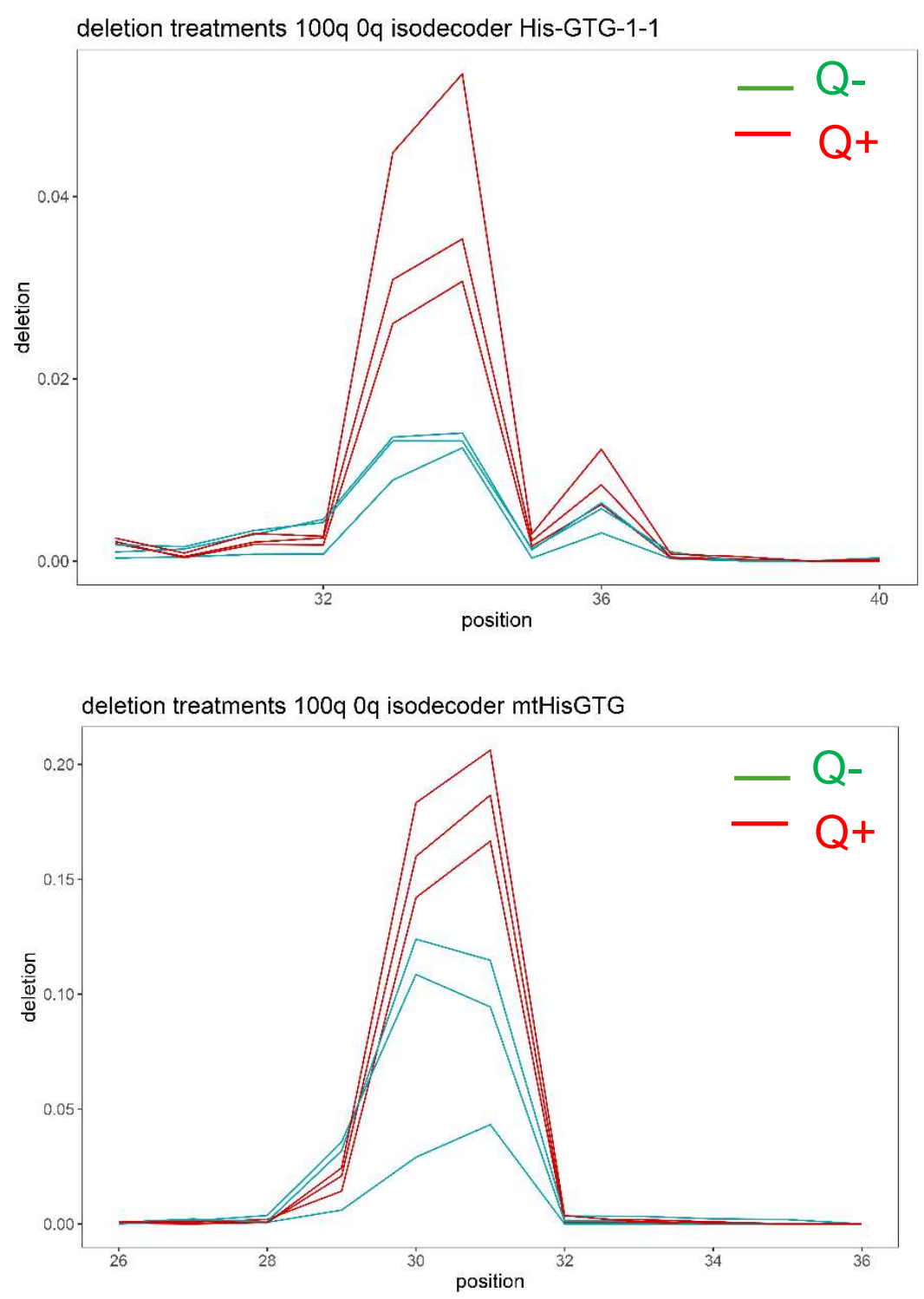

B

Genomic codon usage (per thousand codons)

<u>Chromosome:</u>
Asn: 36.2
Asp: 48.3
His: 25.3
Tyr: 27.6

<u>Mitochrondrion:</u>
Asn: 160
Asp: 63
His: 97
Tyr: 124

Figure S3: Additional data for Figure 3.

(A) PAQS-seq of Q- and Q+ MCF7 cells showing different Q-modification level of cytosolic (top) and mitochondrial (bottom) $tRNA^{His}$. Individual tRNA data from *n*=3 biological replicates are shown.

(B) Genomic codon usage of the 4 amino acids that are decoded by Q-modified tRNAs.

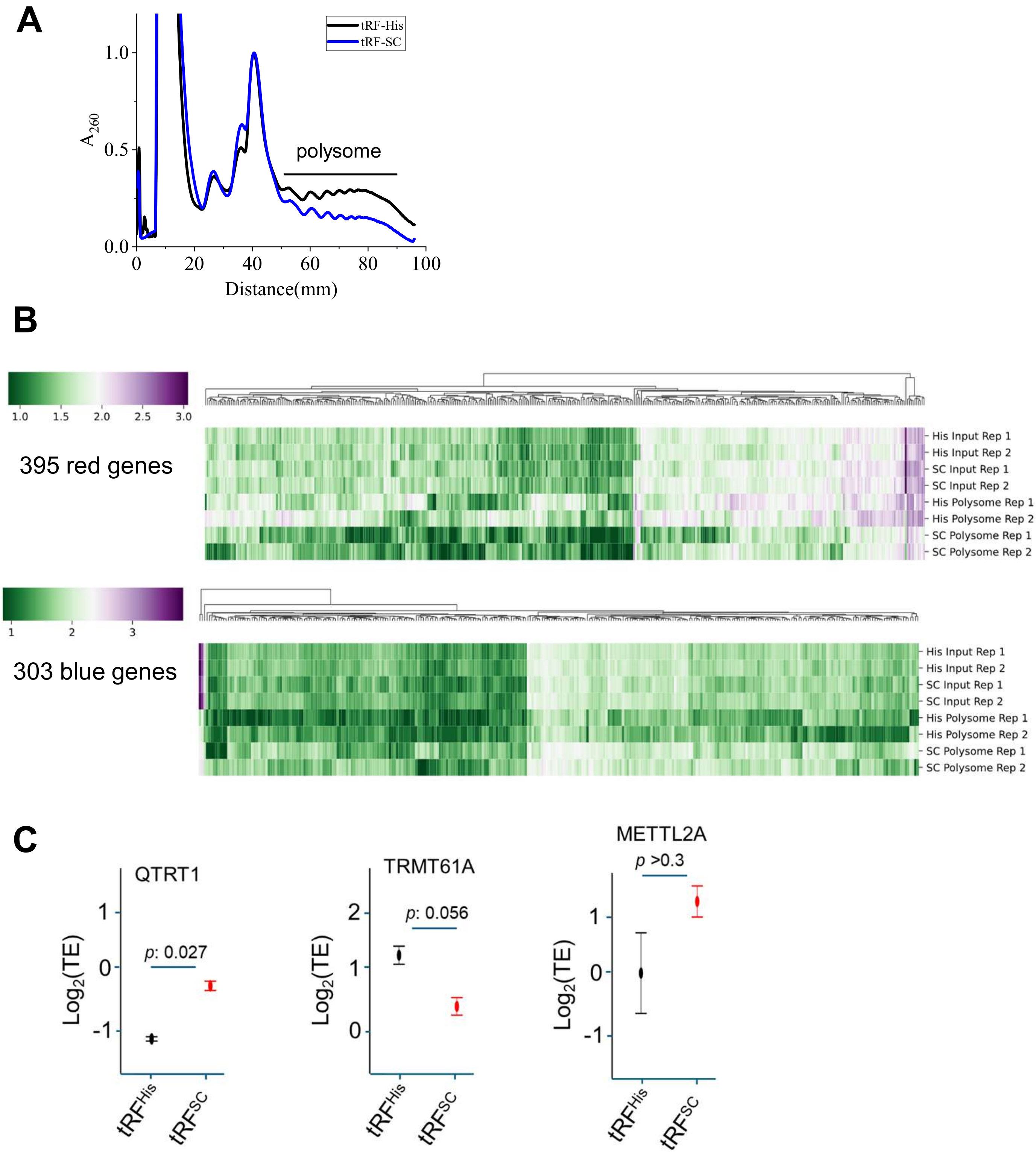

Figure S4: Additional data for Figure 4.

(A) Polysome profile of tRF^{His} or tRF^{SC} transfected, Q+ MCF7 cells. The line indicates the pooled polysome fractions for mRNA sequencing.

(B) Relationship of the 395 "red" genes (top) and 303 "blue" genes in Figure 3B. Red or blue genes have higher or lower TE comparing tRF^{His} over tRF^{SC} transfected cells, respectively.

(C) Comparing translation efficiency of QTRT1 (Q writer), TRMT61A (m^1A58 writer), and METTL2A (m^3C32 writer).

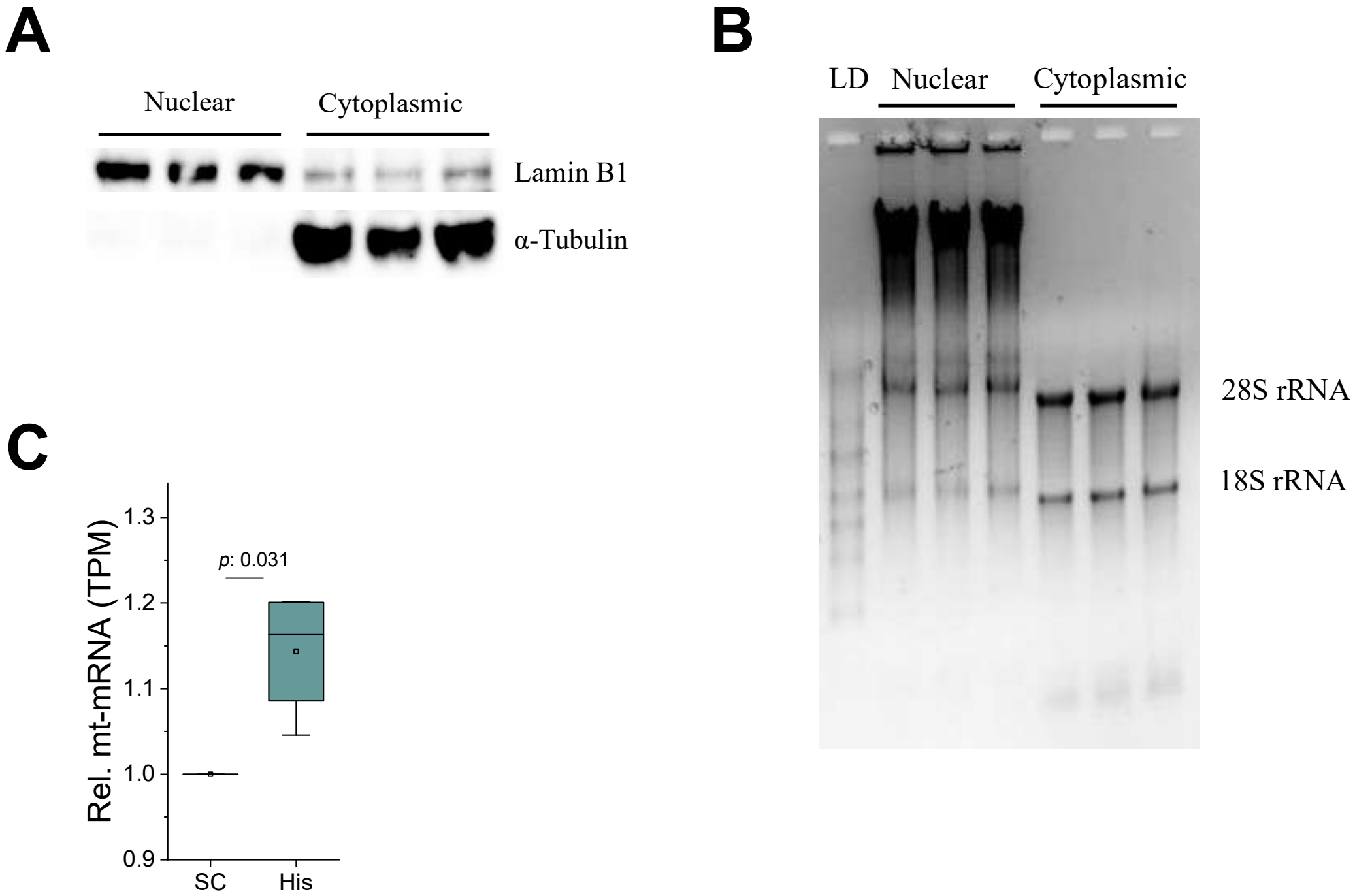

Figure S5: Additional data for Figure 5.

(A) Western blot showing nuclear and cytosolic fractionation.

(B) Total RNA quality check of the nuclear and cytoplasmic fractions used for Northern blot in Fig. 5A.

(C) Comparing the levels of mitochondrial encoded mRNAs for complex III (CytB) and complex IV (COI, COII, COIII) between tRF^{His} and tRF^{SC} transfected MCF7 cells.

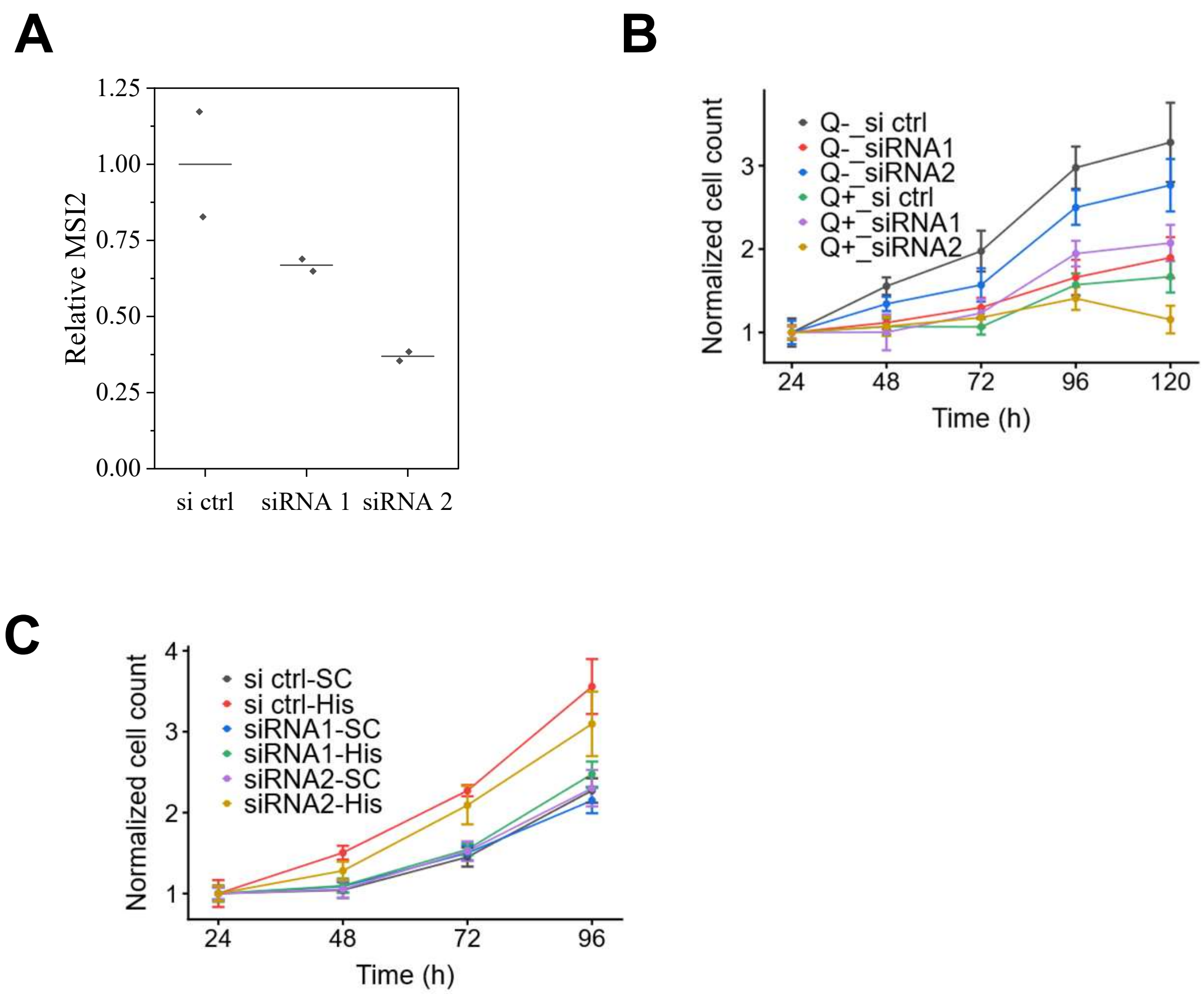

Figure S6 : Additional data for Figure 6.

(A) Quantitation of Msi2 protein knockdown by Western blot in Figure 6A.

(B) Proliferation curve of Q- and Q+ MCF7 cells upon MSI knockdown.

(C) Proliferation curve of Q+ MCF7 cells with MSI2 knockdown and tRF^{His} or tRF^{SC} transfection. Statistical significance is the same as shown in Fig. 6D.
